# Feasibility of internet-based multimodal emotion recognition training in adolescents with and without autism: A pilot study

**DOI:** 10.1016/j.invent.2025.100861

**Published:** 2025-07-10

**Authors:** Nora Choque Olsson, Julia Nordlander Björkman, Rasmus Lackell, Oliver Bergens, Håkan Fischer, Lillian Döllinger, Jan Bergström, Per Carlbring, Petri Laukka

**Affiliations:** a Department of Psychology, Stockholm University, Stockholm, Sweden; b Research Center for Child Mental Development, Chiba University, Chiba, Japan; c School of Psychology, Korea University, Seoul, South Korea; d Department of Psychology, Uppsala University, Uppsala, Sweden

**Keywords:** Autism, Intervention, Multimodal emotion recognition, Training

## Abstract

**Background:**

Research suggests that individuals with autism spectrum disorder (ASD) have difficulties in emotion recognition (ER), which could lead to social difficulties. ER can be enhanced through targeted interventions, but generalization to everyday functioning poses a challenge. Using dynamic multimodal emotional expressions for training may increase similarities to everyday situations. This pilot study investigated the feasibility of internet-based multimodal emotion recognition training (iMERAT) for adolescents with ASD.

**Method:**

Eight adolescents with ASD and nine typically developing (TD) adolescents took part in the iMERAT intervention, which included brief online training sessions conducted each weekday during a 3-week period. Training was performed on dynamic facial, vocal and multimodal emotional expressions, with outcome feedback provided after each response. A survey was conducted to explore participants' experiences of the training. ER was measured pre- and post-training using a multimodal ER test.

**Results:**

Participants reported that the training was moderately difficult, instructions were relatively easy to understand, and the duration of training was appropriate. Content analysis of open-ended responses suggested further adaptations, such as providing more explanations of emotions and further tailoring content and language for adolescents. ER increased from pre- to post-intervention, with large effect sizes for both ASD and TD adolescents.

**Conclusion:**

Results suggest that the iMERAT intervention is feasible for adolescents with ASD. Gains in ER ability were observed, but the small sample size and lack of a control group render these findings tentative. Further research is required to assess the effectiveness of the iMERAT and possible impact on broader social skills.

## Highlights

• iMERAT is a feasible internet-based emotion recognition training for youth with ASD.
• Instructions were relatively clear and the task was not too hard or time-consuming.
• Qualitative feedback suggested that more adaptations to the training may be needed.
• Emotion recognition increased post-training, with large effect sizes.
• Small sample and lack of control group limit conclusions about effectiveness.

## 1 Introduction

Emotion recognition (ER) refers to the ability to accurately perceive, identify, and label emotions expressed by others through nonverbal cues. Such cues may include facial expressions, vocal cues, and body language (e.g., Keltner et al., 2019; Laukka et al., 2021; Zupan and Eskritt, 2024). Autism Spectrum Disorder (ASD) is characterized by persistent difficulties with social interaction and communication, often accompanied by stereotyped and repetitive behaviors and sensory abnormalities (Lord et al., 2020). Difficulties with social interaction include a lack of reciprocity in social communication, poor understanding of nonverbal behavior, and challenges in forming and maintaining relationships (Restoy et al., 2024; Segal et al., 2017). Difficulties with ER in autistic individuals are well-documented (e.g., Costescu et al., 2019; Griffiths et al., 2019; Leung et al., 2023; Loth et al., 2018; Philip et al., 2010; Fridenson-Hayo et al., 2016; Law Smith et al., 2010), and reviews suggest a prevalence ranging from 50 to 85 % across various studies (Leung et al., 2022; Masoomi et al., 2025; Uljarevic and Hamilton, 2013; Yeung, 2022). Lower ER ability has also been shown to correlate with lower levels of social interaction (Hudepohl et al., 2015).

ER impairment in autism has been attributed to general challenges in theory of mind (i.e., the ability to understanding others' feelings, beliefs, and thoughts; Baron-Cohen et al., 1985), and more specifically to challenges in modulating eye contact (Tanaka and Sung, 2016) and attentional and cognitive processing of emotional faces (Black et al., 2017; Lozier et al., 2014; Sasson, 2006; Zhi et al., 2021). Frequent failures to correctly identify others' emotions in social situations can contribute to increased incidence of depression and anxiety, and impaired academic performance (Pickett et al., 2004; Reed et al., 2023; Nyquist and Luebbe, 2020; Warnes et al., 2005; Zhang et al., 2024).

Several training programs have been developed to enhance ER ability based on facial expressions for individuals with autism (e.g., Bölte et al., 2002; Grossard et al., 2020; Petrovska and Trajkovski, 2019; Rice et al., 2015; Russo-Ponsaran et al., 2016; Thomeer et al., 2015). Efforts have also focused on increasing the accessibility and effectiveness of these programs through mobile applications and gamification (e.g., Egger et al., 2018; Fridenson-Hayo et al., 2017; Garcia-Garcia et al., 2022; Zhang et al., 2019). However, most previous training programs have been unimodal and focused on mainly visual-only stimuli (Zhang et al., 2021; Zhi et al., 2021), which may limit their ecological validity (Zhang et al., 2020). Multimodal interventions, which include a broader range of nonverbal signals, may more closely resemble real-life scenarios and could potentially enhance the generalization of training to new contexts (Berggren et al., 2018; Fridenson-Hayo et al., 2017; Thomeer et al., 2015). In this pilot study, we therefore investigated the feasibility of internet-based multimodal ER training (including dynamic facial and vocal emotion expressions) for adolescents with ASD.

When delivering interventions in an internet-based format, treatment adherence has been shown to increase when patients are content with the intervention and delivery mode (Beatty and Binnion, 2016). Pellicano et al. (2014) emphasized the importance of including autistic individuals in the development of interventions to ensure they reflect their needs and preferences. Despite this, little is known about how adolescents experience internet-based ER training, although this could offer valuable insights for improving such programs. Addressing this gap, the present study focuses on autistic adolescents' views of an online ER training program and its preliminary impact on their ER abilities.

The aim of the present study was thus to investigate the feasibility of an internet-based multimodal ER training program (iMERAT) in adolescents with ASD, and also for typically developing (TD) adolescents, addressing the following main research questions:

1) What were the participants' experiences with the iMERAT training in both groups, and what suggestions did they have for improving the program?
2) Does ER ability change from pre- to post-training among autistic adolescents and TD adolescents?

## 2 Method and analysis

### 2.1 Participants

Participants were recruited for two groups: adolescents with ASD diagnosis and TD adolescents aged 13–17 years. Inclusion criteria for the ASD group were: a prior ASD diagnosis (according to ICD-10: F84.0, F84.1, F84.5, or F84.9; World Health Organization, 2004) ascertained by multidisciplinary assessment teams, and an intellectual assessment (IQ ≥ 70) confirmed by a copy of a written neuropsychiatric evaluation. Participants also needed to have access to a computer and internet connection. Exclusion criteria for the autistic group were: a history of self-harm, conduct disorder (F91), emotionally unstable personality disorder (F60.3), or schizophrenia or psychotic disorders (F20–29). Inclusion criteria for the TD group were: typical development, defined as the absence of any autism diagnoses or severe psychiatric diagnosis, and access to a computer and internet connection. The same exclusion criteria to the TD group as to the autistic group were applied, along with any ongoing treatment or medication. Written informed consent was obtained from each participant and their parents after the study's aims and procedures had been fully explained. The study was approved by the Swedish Ethical Review Authority (reference number 2022-04397-01). Data collection took place during the spring of 2023 and spring of 2024.

A total of 8 autistic adolescents and 9 TD adolescents participated in the study. Of these participants, 7 autistic adolescents and 6 TD adolescents completed both the pre- and post-assessments of ER and were thus included in the statistical analysis. The reason for dropping out in both groups was other daily activities such as schoolwork or leisure activities. In the quantitative part of the survey, response rates among TD adolescents varied by question but 8 participants responded to all questions. Among autistic adolescents, 6 participants responded to the quantitative evaluation survey. The open-ended questions were answered by 6 autistic participants and 7 TD participants.

Participants were recruited through advertisements in schools and psychiatric clinics in the Stockholm area, as well as via social media. Among autistic adolescents, 50 % were girls and 50 % were boys. Among the TD participants, 56 % were girls, 33 % were boys, and 11 % identified as “other”. The mean age in the autistic group was 15.8 years (SD = 0.97), while the mean age of participants without autism was 14.2 years (SD = 1.51). In the autistic group, one participant had co-occurrent ADD (F90.0), unspecified anxiety (F419), and recurrent depressive episodes (F33.9). In the TD group, there was one person with a language disorder (F802b), one person with dyslexia (F81.0), and one participant with unspecified anxiety (F419) according to the ICD-10 (World Health Organization, 2004).

### 2.2 Intervention: internet-based multimodal emotion recognition training (iMERAT)

The iMERAT intervention consisted of training ER separately for audio, video and audio-video stimuli. The participants trained to recognize 12 emotions (anger, anxiety, despair, disgust, fear, interest, joy, pleasure, pride, relief, irritation, and sadness) expressed through vocal expressions (audio-only), facial expressions (video-only) and multimodal expressions (audio-video). The training material contained 144 items (brief video clips) from the Geneva Multimodal Emotion Portrayal (Gemep) database (Bänziger et al., 2012), with 72 items randomly selected for each training session (24 audio-only, 24 video-only, and 24 multimodal items). Audio-only items feature a voice speaking a pseudo-language, video-only items show dynamic facial expressions, and multimodal items present voice and corresponding dynamic facial expressions together. During a training session, participants judged which emotion was expressed in each item by selecting one of 12 alternatives, corresponding to the intended emotions. Feedback was provided after each response, indicating whether the chosen emotion matched the intended emotion or not through a thumbs-up or thumbs-down image. Practice with feedback has been proposed to be the most effective component for training ER in previous studies (e.g., Blanch-Hartigan et al., 2012). There was no time limit for judging the items, but each item could only be viewed once during a session. The duration of the items was approximately 2–4 s, and examples are provided in Fig. S1 in the supplemental material.

Participants were instructed to conduct one training session on each weekday for a 3-week period. The training was estimated to take approximately 20 min per session, but this varied among participants depending on the time spent on each item. Participants were able to choose the timing for their daily training sessions, and daily reminder text messages were sent at agreed times if requested by the participant or their parent. The iMERAT intervention and the emotion recognition assessments and survey (described below) were all administered via the Qualtrics platform (Qualtrics, Provo, UT).

The iMERAT intervention was adapted from previous ER training studies with non-autistic adults (Döllinger et al., 2021, Döllinger et al., 2023), where a similar training procedure led to large pre-to-post improvements for the training group compared to controls who did not receive training. The procedures and the language of the instructions were adapted for adolescents in the current study, and these adaptations were reviewed by an independent autistic adolescent, who also considered the duration of the training as adequate. Written descriptions of the emotions and scenarios where the emotion might be experienced were provided at the start of each training session. These descriptions were adapted for adolescents using more accessible language (see Table S1 in the supplemental material).

### 2.3 Materials and instruments

#### 2.3.1 Feasibility questionnaire

A survey including both quantitative and qualitative items (Creswell and Plano Clark, 2011; Fetters et al., 2013) was used to collect participant feedback on the iMERAT training. Responses were collected within 2 to 3 weeks after participants finalized the training. The survey was in Swedish and all questions and answers were translated to English for this publication. In the first five questions the participants rated their experience of the training program's difficulty, instructions, effectiveness, time consumption, and overall experience on scales ranging from 1 (min) to 7 (max). Additionally, participants reported how many minutes they spent on the study as a whole and the number of minutes per day spent on the training alone. Participation in the study did not require any other daily activities beyond the training, and the question about study participation aimed to capture unexpected time expenditures. The exact wording of the questions is presented in the results section.

The next five questions were open-ended. Here, participants were asked to write about specific aspects of their experience of using the program, including whether the instructions and materials were clear, how participants handled any difficulties, what they found positive and negative about the training, what changes they would prefer to be made, and whether they had any additional comments or had encountered any trouble or uncomfortable situations during the training.

An independent adolescent with ASD reviewed the questions in the survey prior to the pilot study.

#### 2.3.2 Emotion recognition assessment (ERAM)

The ERAM (Emotion Recognition Assessment in Multiple Modalities) test (Laukka et al., 2021) was used to measure ER before and after the training. It consists of 72 items (brief video clips) wherein adult actors portray 12 emotions (anxiety, despair, disgust, anger, interest, irritation, joy, fear, pleasure, pride, relief, and sadness) and each emotion is presented in three modalities (24 audio-only, 24 video-only, and 24 multimodal items). The items included in ERAM test are selected from the Gemep database (Bänziger et al., 2012), but no items from ERAM were used in the iMERAT intervention to avoid learning effects. Participants judged the emotion conveyed in each item by selecting one alternative from a list of 12 alternatives (same as the intended emotions). The test took about 20 min to complete, but there was no time limit for responding to each item. The test results include the number of correctly identified items, with results broken down by modality and emotion (Laukka et al., 2021). Previous studies on the psychometric properties of ERAM have shown internal consistency ranging from acceptable to good (Döllinger et al., 2021; Laukka et al., 2021). Written descriptions of each emotion and scenarios where the emotion might be experienced were provided at the start of each test occasion (see Table S2 in the supplemental material).

### 2.4 Data analysis

#### 2.4.1 Feasibility questionnaire

Participants' responses to the fixed response questions were analyzed descriptively. The frequency of responses was tallied, and the relative frequency of each option was calculated. Mean scores for each group were also calculated for each question. Questions 6 and 7 were presented similarly, but responses were reported in minutes.

Participants' responses to the open questions were analyzed qualitatively, using content analysis (Krippendorff, 2013) with an inductive approach. The following steps were followed during the analysis:

1) Sorting data into meaningful units: The author JNB read all responses and condensed all reflections, comments, and suggestions regarding the program into meaningful units. Participants' phrasing and word choices were retained during this process.
2) Contextualizing data through the researcher's understanding and context: Meaningful units were clustered based on the author's understanding of their content, and units addressing the same aspect of the research question were placed in the same cluster.
3) Relating findings to the research question: Clusters were categorized under subcategories corresponding to different parts of the intervention, and then into broader categories related to different aspects of the intervention. As a final step, internal homogeneity and external heterogeneity were checked by authors JNB and NCO by reviewing the materials, subcategories, and categories.

#### 2.4.2 Emotion recognition assessment (ERAM)

Differences in ERAM-scores (total score, video-only, audio-only and multimodal) from pre- to post-training were analyzed using paired samples *t*-tests in each group. Shapiro-Wilks tests suggested that the pairwise differences did not deviate significantly from a normal distribution. Effect sizes for the differences were calculated using Cohen's *d*. Bonferroni correction was applied to control for multiple testing with a corrected alpha of 0.006 (= 0.05/8). In addition, we evaluated the training effects on ERAM scores using Bayesian paired samples *t*-tests to offer a complementary perspective on the evidence. These analyses used default Cauchy prior scales (*r* = 0.707) to calculate Bayes factors (BF_10_) to estimate the likelihood of training effects (Wagenmakers et al., 2018). According to the null hypothesis (H_0_) we expected no difference in ER from pre- to post-intervention, whereas the two-sided alternative hypothesis (H_1_) was that the differences would be different from 0.

## 3 Results

### 3.1 Feasibility questionnaire: quantitative findings

Table 1 presents the frequencies of different response options and the mean values for the responses to the quantitative survey questions. The overall impression of the training was positive for both groups, with only one participant per group reporting a negative impression. On average, participants in both groups reported that they found the training useful for improving emotion recognition, although there were also participants who gave opposite ratings. The training seemed to have an appropriate level of difficulty, with most ratings around the middle of the scale for both groups, and participants in both groups also reported that the instructions were easy to understand. Only one participant in each group found the instructions difficult to comprehend. Finally, participants reported that the task required an appropriate amount of time, and most participants spent between 10 and 20 min on the training as intended, although there were also participants who spent less or more time.

**Table 1:** Survey results, including the number, frequency, and mean values for each question, presented separately for the ASD and TD groups.

Question			Score	ASD	TD
1.	How easy or difficult did you find the training (iMERAT)? 1 = “easiest imaginable”, 7 = “most difficult imaginable”		1 2 3 4 5 6 7	0 0 3 (50 %) 1 (17 %) 2 (33 %) 0 0	0 3 (30 %) 0 5 (50 %) 2 (20 %) 0 0
		*M*		3.8	3.6
2.	Were the instructions for the training understandable? 1 = “impossible to understand”, 7 = “very easily understood”		1 2 3 4 5 6 7	0 1 (17 %) 0 0 0 3 (50 %) 2 (33 %)	0 0 1 (11 %) 0 0 4 (44 %) 4 (44 %)
		*M*		5.6	6.1
3.	Did you find the training (iMERAT) useful for improving your emotion recognition? 1 = “it was not useful at all”, 7 = “it was as useful as it could possibly be”		1 2 3 4 5 6 7	1 (17 %) 0 1 (17 %) 1 (17 %) 3 (50 %) 0 0	0 2 (22 %) 2 (22 %) 1 (11 %) 2 (22 %) 2 (22 %) 0
		*M*		3.8	4.0
4.	What did you think about the time spent on the daily training? 1 = “way too time-consuming”, 7 = “just the right amount of time”		1 2 3 4 5 6 7	0 1 (17 %) 1 (17 %) 0 1 (17 %) 2 (33 % 1 (17 %)	0 0 2 (22 %) 0 1 (11 %) 3 (33 %) 3 (33 %)
		*M*		4.8	5.5
5.	What was your overall impression about the training? 1 = “not good at all”, 7 = “as good as I could have imagined”		1 2 3 4 5 6 7	0 1 (17 %) 0 1 (17 %) 2 33 %) 1 (17 %) 1 (17 %)	0 1 (11 %) 0 4 (44 %) 1 (11 %) 0 3 (33 %)
		*M*		4.8	4.9
6.	How much time did you spend daily on the training (iMERAT)? Please answer in minutes.			20 8 10 10 25 20 8	15 11 10 10 9 10
		*M*		14.4 min	8.1 min
7.	How much time did you spend daily on the training (iMERAT), excluding anything but the training itself. Please answer in minutes.			20 7 10 10 25 20	15 10 10 10 3 20 10 30
		*M*		15.3 min	13.5 min

Note: iMERAT = Internet-based multimodal emotion recognition training; ASD = autism spectrum disorder; TD = typically developing; M = mean.

### 3.2 Feasibility questionnaire: qualitative findings

The qualitative analysis of the participants' responses to the open questions in the survey revealed three main categories (“structure”, “content” and “other”), which were further divided into seven subcategories. Table 2 presents all responses and examples of quotes categorized into categories and subcategories.

**Table 2:** Categories and subcategories identified in the qualitative analysis.

Category	Sub-category	Examples of quotes	
		ASD group	TD group
Structure	Instructions	“The instructions were very simple and easy to understand” (P1) “Easy, simple, ok, an adult helped me by explaining them, not easy to memorize” (P10)	“Very easily understood, so you always understood what was going to happen and what you were supposed to do” (P2) “It is hard to memorize the different emotions, but I still managed” (P8)
	Feedback	“Nice to see results, let me know if I was right or wrong” (P12)	“Good, confirmation whether you were right or wrong after an expression” (P4)
Content	Words describing emotions	“Many of the options felt like the exact same thing, making it hard to know which option was correct” (P9)“ … some of the emotions you could choose from were hard to understand, like disgust and anxiety” (P1)	“Felt wrong, I think several answers are correct for some expressions, some were hard to understand” (P2)
	Training materials	“The clips with audio and video were the easiest to understand, annoying that they didn't speak Swedish, a language I don't understand” (P11) “Same clips were played many times, making it very boring, and I remembered the correct answers for the different clips” (P12)	“Good with different people acting, memorized videos, learnt what people did which emotions, good with different videos. Several clips were repeated during the training” (P4) “It was very hard to recognize several emotions, especially when it was just audio. Many voices sounded very similar, making it hard to find an emotion that matched” (P2)
	Suggestions for improvement	“… maybe replace these [emotion] words with easier-to-understand ones” (P1)	“Yes, make the emotions clearer” (P5) “More variety in who played which roles…” (P4) “The actors in the clips should speak an existing language” (P6)
Other	Technology	“Good that it was easy to do on the phone” (P9) “Nothing was bad, but the videos weren't fully adapted for viewing on a mobile device since you had to move the picture to see everything” (P9)	“... sometimes the sound didn't work … and then I just had to guess” (P5) “That you only got to watch or listen once and didn't get another chance to watch/listen to it again. This made it quite difficult to determine which emotion it was” (P8)
	Reflections	“It teaches you to distinguish similar emotions [e.g., anxiety and despair], which has helped me in social interactions with others” (P7)	“Educational, you learn” (P6)

Note: P = participant; ASD = autism spectrum disorder; TD = typically developing.

#### 3.2.1 Structure

This category included participants' experiences of the training design*.* It is divided into the subcategories *Instructions* and *Feedback*.

##### 3.2.1.1 Instructions

Among the six autistic participants, three mentioned their perceptions of the instructions. Two of them found the instructions adequate and easy to understand, while one participant needed help from an adult. Two participants found it difficult to remember the descriptions of the different emotions presented at the beginning of the training. The majority of TD adolescents who completed the survey indicated that they found the training instructions to be clear. Several TD participants also mentioned difficulties in remembering how the different emotions were described before starting the training.

##### 3.2.1.2 Feedback

Two autistic participants mentioned that they appreciated the feedback, with one stating that the feedback was a particularly good aspect of the training. Two of the TD participants also mentioned that they appreciated receiving feedback after each item during training.

#### 3.2.2 Content

This category included participants' experiences of the content and materials of the training. It is divided into the subcategories *Words describing emotions*, *Training materials*, and *Suggestions for improvement*.

##### 3.2.2.1 Words describing emotions

Three autistic participants found the words used to describe emotions difficult to understand (e.g., disgust and anxiety), and suggested a possible improvement using words that are easier to understand. All TD participants provided feedback on the words used to describe emotions in the intervention. The majority of TD participants felt that the descriptions given at the start of the training did not align with their prior understanding of the emotions and corresponding words.

##### 3.2.2.2 Training materials

In the group with ASD, two participants highlighted aspects they liked about the training materials, such as having multiple actors portray different emotions and the multimodal nature of the training. Regarding the audio and video clips, two autistic participants disliked that the actors did not speak Swedish. The majority of TD participants provided varied feedback on the audio and video clips used in the training. Two TD participants felt that the variety of training clips was too narrow. Two TD participants found the audio and video clips difficult to interpret, noting challenges in recognizing several emotions.

##### 3.2.2.3 Suggestions for improvement

One autistic participant suggested that the emotion words could be replaced with ones that are easier to understand. Two TD participants wanted the audio and video clips to be clearer. Additionally, two TD participants wanted more variety in the actors in the clips. Finally, two TD participants also wished for simpler explanations of the emotions and suggested that the actors in the clips should speak an existing language.

#### 3.2.3 Other

Other themes arose that did not fit the categories. The *Other* category includes the subcategories *Technology* and *Reflections*.

##### 3.2.3.1 Technology

One of the autistic participants made two comments regarding the use of mobile devices to run the training program. Three TD participants described technical difficulties in the survey. Two participants mentioned that the sound occasionally did not work and two participants noted that it was not possible to watch or listen to the clips again.

##### 3.2.3.2 Reflections

Three autistic participants provided general reflections on the training. Two of them found it beneficial to train on recognizing emotions and learn to distinguish similar emotions (e.g., anxiety and despair), which could facilitate social interactions with others. The third emphasized the importance of doing the training during periods when they had time to dedicate to it, otherwise the training could become a hindrance. Two TD participants mentioned that they found the training informative.

### 3.3 Changes in ER ability from pre- to post-intervention

Emotion recognition rates (proportion of correct responses) at pre- and post-training are displayed in Table 3 for adolescents with ASD and in Table 4 for TD adolescents. Both groups showed large increases (Cohens's *d* = 1.06–1.56) in ER from pre- to post-training for overall scores and the audio-only and multimodal conditions, and moderate to large increases (Cohen's *d* = 0.71–0.83) for the video-only condition.

**Table 3:** Emotion recognition accuracy at pre- and post-training for the ASD group (*n* = 7) and results from frequentist and Bayesian paired samples *t*-tests.

ERAM	Pre M (SD)	Post M (SD)	*t* value	df	*p* value	Cohen's *d*	95 % CI	BF_10_
Total	0.46 (0.12)	0.63 (0.11)	5.88	6	0.001⁎⁎	1.43	[0.32, 2.49]	38.87^v^
Video	0.48 (0.14)	0.64 (0.18)	7.12	6	<0.001⁎⁎	0.71	[−0.15, 1.52]	87.75^v^
Audio	0.46 (0.11)	0.61 (0.11)	4.75	6	0.003⁎⁎	1.38	[0.29, 2.42]	16.52^s^
Multimodal	0.44 (0.14)	0.64 (0.12)	3.23	6	0.018⁎	1.56	[0.40, 2.68]	4.30^m^

*Note*. Cohen's *d* corrected for correlation between observations. Bayes factor interpretation: v = very strong evidence for H_1_; s = strong evidence for H_1_; m = moderate evidence for H_1_.

⁎ Significant results of the paired samples *t*-test (alpha = 0.05).

⁎⁎ Significant results after Bonferroni adjustments (alpha = 0.006).

**Table 4:** Emotion recognition accuracy at pre- and post-training for the TD group (*n* = 6) and results from frequentist and Bayesian paired samples *t*-tests.

ERAM	Pre M (SD)	Post M (SD)	*t* value	df	*p* value	Cohen's *d*	95 % CI	BF_10_
Total	0.51 (0.07)	0.66 (0.13)	3.81	5	0.012⁎	1.30	[0.15, 2.39]	5.84^m^
Video	0.56 (0.15)	0.67 (0.09)	1.72	5	0.146⁎⁎	0.83	[−0.14, 1.74]	0.97^a0^
Audio	0.46 (0.06)	0.64 (0.15)	4.11	5	0.009⁎	1.06	[0.004, 2.05]	7.31^m^
Multimodal	0.50 (0.07)	0.68 (0.15)	2.85	5	0.036⁎	1.48	[0.25, 2.64]	2.66^a^

*Note*. Cohen's *d* corrected for correlation between observations. Bayes factor interpretation: m = moderate evidence for H_1_; a = anecdotal evidence for H_1_; a0 = anecdotal evidence for H_0_.

⁎ Significant results of the paired samples *t*-test (alpha = 0.05).

⁎⁎ Significant results after Bonferroni adjustments (alpha = 0.006).

For the ASD group, pairwise *t*-tests indicated that the increase was statistically significant for overall scores and the video-only and audio-only conditions, but not for the multimodal condition, after correction for multiple testing (see Table 3). The results from the Bayesian analyses yielded a similar picture, with Bayes factors (BF_10_) for overall scores and video-only scores showing very strong evidence in favor of H_1_. For example, for the overall scores (BF_10_ = 38.87) the results indicated that the observed data was approximately 39 times more likely under H_1_ than H_0_. Audio-only scores showed strong, and multimodal scores moderate, evidence in favor of H_1_ (see Table 3).

For the TD group, pairwise *t*-tests indicated that the pre-post increases did not reach statistical significance after correction for multiple testing (see Table 4). The Bayesian analyses showed that there was moderate evidence for H_1_ for both overall and audio-only scores. For example, the Bayes factor for overall scores (BF_10_ = 5.84) indicated that the observed data was approximately 6 times more likely under H_1_ than H_0._ The results for video-only and multimodal conditions, however, provided only anecdotal evidence.

Group differences were not analyzed statistically because of the small number of participants in each group. Anecdotally, it can be noted that while both groups had similar scores in the audio-only condition, the ASD group displayed lower ER in the video-only and multimodal conditions, especially before training.

## 4 Discussion

The aim of this study was to evaluate the feasibility of an internet-based multimodal emotion recognition training program (iMERAT) for autistic adolescents. While most previous intervention studies have focused on unimodal expressions, often static pictures of facial expressions, the iMERAT instead included dynamic stimuli where emotions were expressed through both facial and vocal expressions, for a more ecologically valid task. Feasibility survey results indicated that the training had a suitable level of difficulty, relatively clear instructions, and was not overly time-consuming. Qualitative feedback also suggested areas for further adjustments, such as clearer explanation of emotions and more tailored content, which might increase the program's usability. Assessment of ER ability pre- and post-training using the multimodal ERAM test (Laukka et al., 2021) suggested that ER ability increased after training, with stronger evidence for the ASD group compared to TD adolescents.

Although autistic adolescents in general reported positive impressions of the iMERAT training in their questionnaire responses, opinions also varied, reflecting the group's heterogeneity (Mirzaei et al., 2022). Autistic adolescents particularly noted difficulty with the emotion descriptions, suggesting these were not sufficiently adapted to their needs. This could stem from challenges in reading comprehension involving social cognition (Brown et al., 2013) or difficulties understanding complex emotions. The training and testing procedures were originally designed for adults (Döllinger et al., 2021; Laukka et al., 2021), but both language and procedures were adapted for adolescents in this study. These adaptations were reviewed by an independent autistic adolescent, but further refinements involving broader feedback from adolescents with autism are recommended (Aarons et al., 2017). This demonstrates the importance of including feedback from the relevant groups to ensure that the developed interventions are tailored to and desired by the target population (Pellicano et al., 2014).

Participants from both groups were critical of the use of pseudo-language in the test and training items, with autistic adolescents raising more frequent objections. Research has suggested individuals with autism may rely on verbal cues for emotion identification (Sprung et al., 2015). However, using real language with verbal cues would reduce the need to decode nonverbal emotional expressions, which is a core goal of the intervention. Retaining pseudo-language while exploring alternative ways to make the task more engaging, such as incorporating gamification techniques, could potentially be beneficial (e.g., Miloff et al., 2015). Participants also suggested greater diversity in training materials. All training sessions of the iMERAT program used the same pool of 144 items from the Gemep database (Bänziger et al., 2012), from which a random selection of 72 items were used for the daily training sessions. While no unintended learning effects were observed since the items included in the ERAM task were distinct from the items that were used for training, a larger set of training items could mitigate potential memorization effects (Mızrak and Oberauer, 2022).

Despite the adolescent's positive general view of the internet-based program's potential, technical difficulties, especially non-functional audio, were reported by several participants. Such issues can frustrate participants, diminish their overall experience, and increase dropout rates in online interventions (North et al., 2000; Welsh et al., 2003; Wentling et al., 2007). Addressing these challenges through user orientation, troubleshooting guidance, support contacts, clearer time commitment estimates and updated technical improvements (e.g., including generative AI) could improve engagement and outcomes (Hentati et al., 2021; Sheridan et al., 2020; Sitzmann et al., 2010). Real-time emotion recognition technologies (Banos et al., 2024) could potentially be used to adapt the content and pacing of the training to the user's level of emotional engagement (Choksi et al., 2024). Perceived relevance and usefulness can further influence engagement in online interventions (Beatty and Binnion, 2016; Ho et al., 2024). Further developments could therefore clarify the goals and possible benefits of the iMERAT intervention, such as how improved ER skills may impact daily life, in order to enhance participant engagement. Finally, we note that although technology-based interventions are generally well received by individuals with ASD (Grynszpan et al., 2014), developers should also take into account possible negative consequences of screen time on well-being (e.g., Costescu et al., 2021; Twenge and Campbell, 2018).

During the iMERAT training, participants received repeated outcome feedback on their responses, allowing for repeated practice with varied presentation forms. This method of training has been shown to be useful for social skills training for adolescents with ASD (e.g., Choque Olsson, 2016). The immediate feedback after each item during training was well-received by participants, aligning with studies highlighting feedback as a critical factor in enhancing ER learning outcomes (Blanch-Hartigan et al., 2012). While research on the impact of feedback on autistic individuals is limited, evidence suggests video feedback enhances learning (e.g., Josol et al., 2022). Later studies have also refuted earlier assumptions that feedback might induce negative feelings about one's abilities upon incorrect responses, at least for TD individuals (Blanch-Hartigan et al., 2012).

We assessed ER ability both before and after the iMERAT training. Frequentist and Bayesian paired samples *t*-tests converged on the finding that ER increased after training for the ASD group. The increase was statistically significant for overall scores and for the video-only and audio-only, but not for the multimodal condition. Likewise, Bayesian analyses showed strong to very strong evidence for H_1_ for all conditions, with the exception that the evidence for H_1_ was moderate for the multimodal condition. This aligns with previous studies showing that training can improve ER in autistic individuals (e.g., Bölte et al., 2002; Grossard et al., 2020; Rice et al., 2015; Thomeer et al., 2015). Pre-post increases in ER were also observed for all conditions in the TD group, in line with previous studies (e.g., Döllinger et al., 2021; Reed et al., 2023), but these increases were not statistically significant after correction for multiple testing despite large effects — most likely due to the small sample size. Bayesian analyses added the information that there was moderate evidence for H_1_ for overall scores and the audio-only condition, but only anecdotal evidence for the other conditions. In conclusion, the statistical evidence for increases in ER ability was stronger for the ASD group than for the TD group. These findings come with several caveats, as discussed below.

We acknowledge that the current study was designed to be a feasibility study, intended to test the novel iMERAT training in a small sample as a preparation for a well-powered and well-controlled future study. Because of the small sample sizes for both the ASD and TD groups, all effect estimates must be considered very preliminary. The small sample size also precluded statistical testing of group differences in ER, although we anecdotally note that the ASD group had lower ER compared to the TD group in line with previous research (e.g., Masoomi et al., 2025). Another important limitation is that we did not include any control group, neither a passive control group that received no intervention, nor an active control group that received sham-training (e.g., Döllinger et al., 2021; Goldberg et al., 2023). This entails that we cannot ensure that increases in ER were caused by the training, rather than by spontaneous improvement due to repeated testing. Previous studies have shown that repeated testing may indeed lead to increased ER scores, but such spontaneous increases are typically smaller than the large effects observed here (e.g., Döllinger et al., 2021) — which makes it less likely that repeated testing alone could have caused the current increases in ER. We also note that it remains to be determined if an increase in scores on the ERAM task reflects an improvement in ER skills that can generalize to everyday social interactions, or if the effects of the training are limited to a particular type of task and setting. Finally, we acknowledge that the lack of follow-up after the training makes it impossible to evaluate whether the acquired skills persist over time. Evidence for sustained training effects at follow-up is limited (Beaumont and Sofronoff, 2008; Zhang et al., 2021), highlighting the need for follow-up ER assessments in future studies. In conclusion, while our preliminary results are promising, they need to be replicated in larger-scale controlled studies, which could include a follow up at, for example, three, six and 12 months. Such studies could also include a variety of ecologically valid measures of ER and other social skills, to determine the broader utility of the iMERAT training for adolescents with ASD.

## 5 Conclusions

In conclusion, this study suggests that iMERAT, a multimodal internet-based ER training program, is feasible for adolescents with ASD. Participant feedback was overall positive, but also highlighted the need for further improvements and adaptations. Results also indicated gains in ER ability, but due to the small sample size and absence of a control group, these findings are tentative. Further research is needed to assess the effectiveness of the iMERAT training and its impact on broader social skills.

## Formatting of funding sources

No funding was received for this study.

## Consent to participate

Informed consent was obtained from all individual participants included in the study.

## Ethical approval

Approval for all study procedures was obtained from the Swedish Ethical Review Authority (reference number 2022-04397-01).

## Declaration of competing interest

The authors declare that they have no known competing financial interests or personal relationships that could have appeared to influence the work reported in this paper.

## Appendix A Supplementary data

Supplementary material Image 1
