# Supplementary material for: Feasibility of internet-based multimodal emotion recognition training in adolescents with and without autism: A pilot study
Source: Internet Interv. 2025 Jul 10;41:100861. doi: 10.1016/j.invent.2025.100861 (PMC12275871; doi:10.1016/j.invent.2025.100861)
Supplement: Supplementary file 1 — Supplementary material [file mmc1.docx]

**Supplemental materials (online)**

**Table S1. Instructions to the iMERAT training**

This is one of your daily exercises to train the ability to recognize emotional expressions. Your task is to watch a series of short audio/video clips of a person showing different emotional expressions. After each recording, you will be asked to choose an emotion from a list. Try to choose the emotion that comes closest to describing the emotional expression demonstrated in the previous audio/video clip.

*This is how the training works:*

You will first be shown three sample recordings. (1) A clip will be shown with both video and audio and after watching it you will be asked to choose an emotion from a list. (2) Then a video recording will be shown, without any audio, and you will again be asked to choose an emotion from a list. (3) Last, a recording with audio and without video will be shown and you will then be asked to choose an emotion from a list again.

*In this study, the concept of emotion is defined as follows:*

*Pride*: Feeling of triumph after a success or a personal achievement

*Anger*: Extreme dissatisfaction caused by someone's unfair or hostile actions

*Joy*: Feeling evoked by a great thing that occurred unexpectedly

*Irritation*: Experiencing dissatisfaction with something or someone while remaining calm

*Disgust*: Dislike caused by an unpleasant object or environment

*Pleasure*: Perceived sense of well-being and sensual pleasure

*Sadness:* Feeling down after loss of a person, place or thing

*Relief*: Feeling of calm at the end or resolution of an uncomfortable, unpleasant or even dangerous situation

*Despair:* Worried by a life problem with no solution, accompanied by an unwillingness to accept the situation

*Interest*: Being fascinated by, or having one's attention captured by, a person or thing

*Fear*: Facing an imminent danger that threatens one's physical well-being

*Anxiety:* Worrying about the consequences of a situation that may be unfavorable to oneself or a loved one.


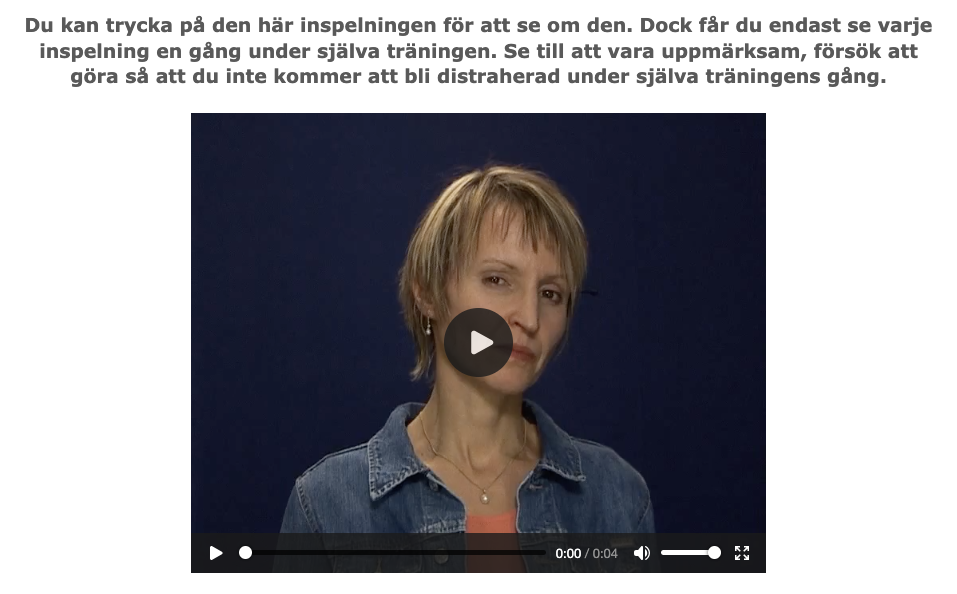


**Figure S1:** Example of the video record. Following presents the translation of the instruction presented above: “You can tap on this recording to watch it again. However, you will only see each recording once during the training itself. Make sure to pay attention, try to make sure that you will not be distracted during the training itself”.

**Table S2. Instructions to the ERAM-test**

Your task is to watch short clips. The clips can contain only audio, only video, or audio and video together. After the clip, we ask you to choose which emotion was shown by the person in the clip. Sometimes it can be difficult to know which emotion was expressed, but please choose the emotion that you think comes closest to describing the emotion shown in the clip.

*In this test, the concept of emotion is defined as follows:*

*Pride*: Feeling of triumph after a success or a personal achievement

*Anger*: Extreme dissatisfaction caused by someone's unfair or hostile actions

*Joy*: Feeling evoked by a great thing that occurred unexpectedly

*Irritation*: Experiencing dissatisfaction with something or someone while remaining calm

*Disgust*: Dislike caused by an unpleasant object or environment

*Pleasure*: Perceived sense of well-being and sensual pleasure

*Sadness:* Feeling down after loss of a person, place or thing

*Relief*: Feeling of calm at the end or resolution of an uncomfortable, unpleasant or even dangerous situation

*Despair:* Worried by a life problem with no solution, accompanied by an unwillingness to accept the situation

*Interest*: Being fascinated by, or having one's attention captured by, a person or thing

*Fear*: Facing an imminent danger that threatens one's physical well-being

*Anxiety:* Worrying about the consequences of a situation that may be unfavorable to oneself or a loved one.

Immediately after watching or listening to the clip, you will be asked to choose between these different emotions. Your task is to choose the emotion that you think best matches the emotion shown in the clip.
